# Item-specific patterns of the Skindex-17 in individuals with different levels of Hidradenitis Suppurativa severity: a network analysis study

**DOI:** 10.3389/fpubh.2023.1284365

**Published:** 2023-10-27

**Authors:** Giulia Raimondi, Tonia Samela, Luca Fania, Damiano Abeni

**Affiliations:** ^1^Clinical Epidemiology Unit, Istituto Dermopatico dell’Immacolata, IDI-IRCCS, Rome, Italy; ^2^Department of Human Sciences, European University of Rome, Rome, Italy; ^3^Clinical Psychology Unit, Istituto Dermopatico dell’Immacolata, IDI-IRCCS, Rome, Italy; ^4^Dermatology Unit, IDI-IRCSS, Rome, Italy

**Keywords:** Hidradenitis Suppurativa, skin disease, Skindex-17, quality of life, network analysis

## Abstract

**Background:**

Recent studies are stressing the idea that the level of Hidradenitis Suppurativa (HS) severity does not always correspond to the same illness load. In fact, it was found that there was no significant association between Skindex-17 and clinical severity of HS, and that some items of the Skindex-17 might be more related to HS severity than others.

**Objective:**

The aim of the current study was to explore the associations between different levels of clinical severity of HS (mild, moderate, severe) and the Skindex-17 single-item responses in a large sample of adults patients.

**Methods:**

A cross-sectional study with a sample include 547 consecutive HS patients, approaching for the first time the specific dermatologic evaluation. Eligible criteria included age ≥ 18  years, HS diagnosis formulated contextually by the same expert clinician, and providing informed consent. All participants completed the Skindex-17 and were evaluated for the disease clinical severity with the International Hidradenitis Suppurativa Severity Score System. A Network Analysis was conducted, which represents a powerful methodological approach in clinical research. It allows to study specific patterns and the structure of complex system in order to better understand how the elements of the system interact with each-other. Three different Network Analyses were conducted for each group of HS clinical severity by including the items of the Skindex-17 as the nodes of each network.

**Results:**

Among 547 patients, 40% (219) were female and mean age was of 32.70 (±11.41). Different patterns among items of the Skindex-17 for the three clinical severity groups emerged, meaning that in each group different items are more crucial than others. The psychosocial subscale of the Skindex-17 is the most relevant when assessing the Quality of Life of individuals with different levels of HS severity, however, with the progression of the disease, individuals place attention also on two different aspects of the symptoms subscale of the Skindex-17, irritation and pain, which in turn perfectly reflect the severity of HS, from a clinical perspective.

**Conclusion:**

These results provide new insights on the association between levels of HS severity and related Quality of Life, measured with the Skindex-17.

## 1. Introduction

Hidradenitis Suppurativa (HS) is one of the most devastating immuno-mediated chronic skin diseases due to its impact on patients’ Quality of Life (QoL) (1). Genetic, environmental, and behavioral factors contribute to the onset and exacerbation of the disease (2–4). Physical pain (5), shame (6), sexual (7), work (5), and social (8) impairments are the main consequences of HS that strongly affect patients’ mental health (9).

To systematically assess the HS illness load, researchers may use disease-specific, specialty-specific, and/or general QoL measures. The results derived from these tools are classified as patient-reported outcomes (PROMS) (10). For example, Kolli et al. using the Short Form Health Survey (SF-36), found significant differences in mean physical component (PCS) and mental component (MCS) of the SF-36 subscales scores, which reflect worse results in terms of QoL, for people with HS compared with healthy controls (11). As stated by the position statement of the European Academy of Dermatology and Venereology task forces on Quality of Life and Patient-Oriented Outcomes (12), among the specialty-specific PROMS, HS illness load has been predominantly assessed by the Dermatology Life Quality Index (DLQI), the Skindex-17, and the Skindex-29 (13–15).

Some recent studies are stressing the idea that the level of disease severity does not always correspond to the same illness load in HS measured with different PROMS (16)—as already highlighted for other chronic skin conditions (17, 18)—and the idea that HS is a very debilitating condition, even at lower levels of clinical severity (15). Regardless of disease severity, as measured with the International Hidradenitis Suppurativa Severity Score System (IHS4) (19), patients with HS often go through the disruption of their personal and social life because of their disease (i.e., especially because of pain, suppuration, and discomfort due to abscesses), as documented by scores obtained in Skindex-17 subscales (15). Sampogna et al. found that there was no significant association between Skindex-17 and clinical severity of HS, measured with IHS4 (15). Moreover, the Authors compared the impact of HS with that of many other skin diseases for all items of the symptom and psychosocial scales of the Skindex-17. HS ranked in the first place for 7 items, second place for 4 items, third place for 3 items and fourth place for 3 items. These results seem to indicate that some items of the Skindex-17 might be more related to HS severity than others, and could explain why no significant association was found as HS severity increased.

Therefore, the aim of the current study was to explore the associations between different levels of clinical severity of HS and the Skindex-17 single-item responses in a large sample of adults diagnosed with HS. Specifically, through a Network Analysis, which represents a powerful methodological approach in clinical research to explore the strength and structure of interactions among variables, we aimed at identifying specific patterns of associations between levels of HS and single items of the Skindex-17, while taking into account the interplay of all items at the same time. Network Analysis allows to study the structure of complex systems and how the variables within a system interact with each other. Since, the measure of QoL in HS patients has led to mixed results (15), the use of Network Analysis can help provide detailed information how each specific symptom (i.e., psychosocial and physical) measured with the Skindex-17 is not only related to different levels of HS severity, but also how it interacts with the other symptoms.

## 2. Materials and methods

### 2.1. Participants

This study is based on the IDI-IRCCS registry of patients with HS. Patients with a new diagnosis of HS or presenting for the first time at the dermatological reference center IDI-IRCCS, in Rome, Italy, with HS were recruited between November 16, 2015 and December 21, 2022. Inclusion and exclusion criteria for this cross-sectional study are described in detail elsewhere (20). The study was approved by the Institutional Ethical Committee of IDIIRCCS (459/1, 9 November 2015). All patients voluntarily signed an informed consent and anonymity was ensured by assigning a code in order to replace patients’ names and identifying information. Referring to the current institutional HS registry, patients’ mean age was of 31.69 (±12.48) and 432 patients (62.2) were women.

### 2.2. Measures

#### 2.2.1. Clinical severity: International Hidradenitis Suppurativa Severity Score System

The International Hidradenitis Suppurativa Severity Score (IHS4) (19) is a scoring system obtained by the number of nodules (i.e., an abnormal amount of solid tissue), plus the number of abscesses (i.e., a skin lesion filled with pus) (multiplied by 2) plus the number of draining tunnels (i.e., narrow tunnel-like structures which are formed under the skin) (multiplied by 4). HS is defined as mild with scores ≤3, moderate with scores ≥4 and ≤10, severe with scores ≥11. The IHS4 is the measure most used world-wide to assess the severity of HS. This scoring system was created by including patients from 11 centers in 6 different countries, providing support to the external validity of the tool, and was approved by the European Hidradenitis Suppurativa Foundation (EHSF) (19).

#### 2.2.2. Quality of life

The Skindex17 (21) is the reduced version of the Skindex-29 and measures the impact of skin conditions on patients’ QoL. It consists of 17 items which define 2 subscales, Psychosocial and Symptoms. Items are rated on a 3-point scale (i.e., 0 = “*Never*,” 1 = “*Rarely/Sometimes*,” 2 = “*Often/Always*”), with higher scores indicating a higher impact on QoL. Scale scores were transformed to a linear scale, with scores ranging from 0 to 100. The Skindex-17 was chosen because it takes into account psychological and social aspects of everyday-life not addressed by other instruments assessing Health Related Quality of Life (HRQoL) characteristics (22). Moreover, the Skindex-17 was created by using analyses from the Item Response Theory (IRT), which allows to estimate the specific contribution of each item to test score (21).

### 2.3. Statistical analyses

All the analyses were performed with the packages “*Bootnet*” (version 1.5), “*Networktools*” (version 1.5), “*ggplot2*” (version 3.4.2), “*qgraph*” (version 1.9.4), and “*igraph*” (version 1.4.2) for R studio, and with the Statistical Package for the Social Sciences, SPSS 25 (23). SPSS is one of the most used software for conducting analyses in clinical contexts, and R provides the most up-to-date packages to perform Network Analyses, since it is an open-source software constantly updated.

Firstly, all patients with missing data either for IHS4 or Skindex-17 were removed.

Network analysis is a statistical methodology that allows to study the pathways and their strengths of the variables considered in a model (24). Each variable entered in the model is considered a node, and through the network analysis, ties between nodes, which are called **Edges**, are explored. In our analyses, the items of the Skindex-17 were the nodes of our model. To assess whether different patterns of the Skindex-17 items were more associated to specific levels of HS severity, the sample was split into 3 groups (Group 1: mild HS; Group 2: moderate HS; Group 3: severe HS) by using the published cut-off scores (mild HS ≤3; ≥4 moderate HS ≤10; severe HS ≥11) of the IHS4. To assess the paths between all nodes, statistical indices called measure of centrality were used. Specifically, **Betweenness**, which refers to the number of shortest edges a node shares with the other nodes in the network; **Closeness**, which refers to the inverse sum of the edges between a node and all the other nodes (e.g., if one item has high closeness then it would be directly connected to the other variables in the network); **Strength**, which refers to the sum of the strengths of each path between a node and all the other nodes. In sum, variables that have higher centrality indices are more central to the network and have shorter and stronger associations with the other variables.

A “least absolute shrinkage and selection operator” (LASSO) (25) was used. The LASSO estimates partial correlation in the network and small weak edges are reduced to zero. It is important to note that the absence of edges does not indicate that there is no relationship between nodes, but the aim of the LASSO is to exclude spurious edges. The LASSO also yields a more parsimonious graph, reflecting only the most important empirical relationships between the variables. Therefore, the LASSO was chosen because it helps estimating models that are more easily interpretable. The LASSO produces a collection of networks rather than a single network, therefore to select the most adequate network model the Extended Bayesian Information Criterion (EBIC) was used (26). Statistical research (27) has proved that the EBIC works well in the estimation of the Gaussian Graphical Model (GGM) (28), which allows edges to be interpreted as partial correlation coefficients and can be used also for ordinal data (29), as the Skindex-17 item-response scale.

All analyses were conducted using a bootstrap approach to calculate 95% confidence intervals (CI) for the edge values (30).

## 3. Results

The registry contained information on 694 persons with HS. After excluding patients with missing data, the final sample included 547 individuals: 100 (18.3%) of them with mild HS, 181 (33.1%) patients having moderate HS, and 266 (48.6%) with severe HS. Among the 547 patients, 40% (219) were female and mean age was of 32.70 (±11.41). Descriptive information on the sample is summarized in Table 1.

**Table 1:** Description of the main characteristics of the study population (*N* = 547).

Variables	No. | M	% | (SD)
Age *M* (SD)	32.70	(11.41)
**Gender *N*/%**		
Men	219	40%
Women	328	60%
**Age onset HS** *M* (SD)	23.47	(43.07)
**Age first diagnosis** *M* (SD)	30.79	42.93
**Sk-PsychoSocial** *M* (SD)	48.14	(28.44)
2. Work/hobbies		
3. Social life		
4. Depressed		
5. Stay home		
7. Closeness		
8. Do things alone		
10. Show affection		
12. Embarrassed		
13. Frustrated		
14. Be with people		
15. Humiliated		
17. Sex life		
**Sk-Symptoms** *M* (SD)	62.21	(23.01)
1. Hurts		
6. Itches		
9. Water bothers		
11. Irritated		
16. Bleeds		

***Note.*** *No. = Number; M = Mean; % = Percentage; SD = Standard Deviation; Sk-PsychoSocial = Psychosocial subscale of the Skindex-17; Sk-Symptoms = Symptoms subscale of the Skindex-17*.

**Figure 1:**
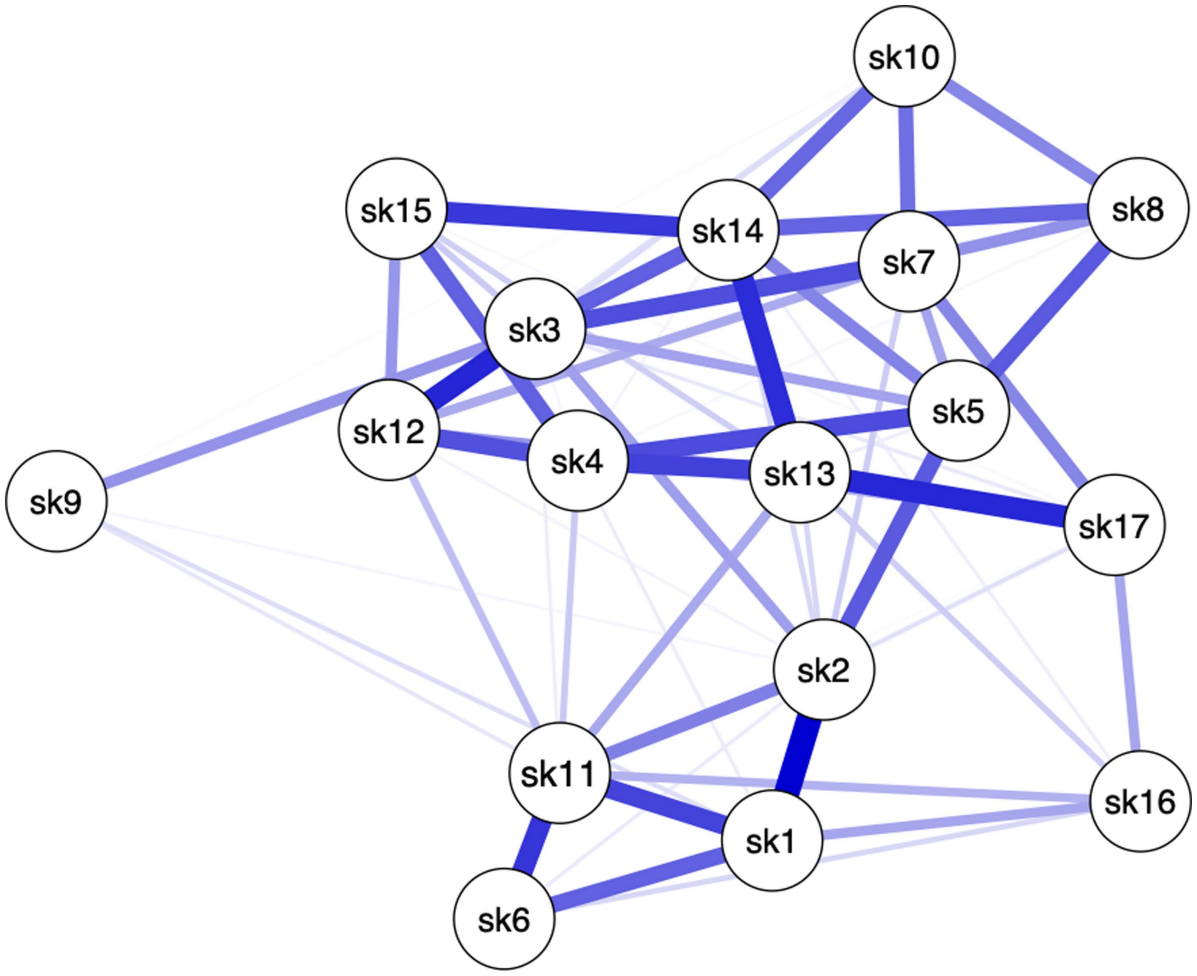
Network of Group 1: mild HS.

**Figure 2:**
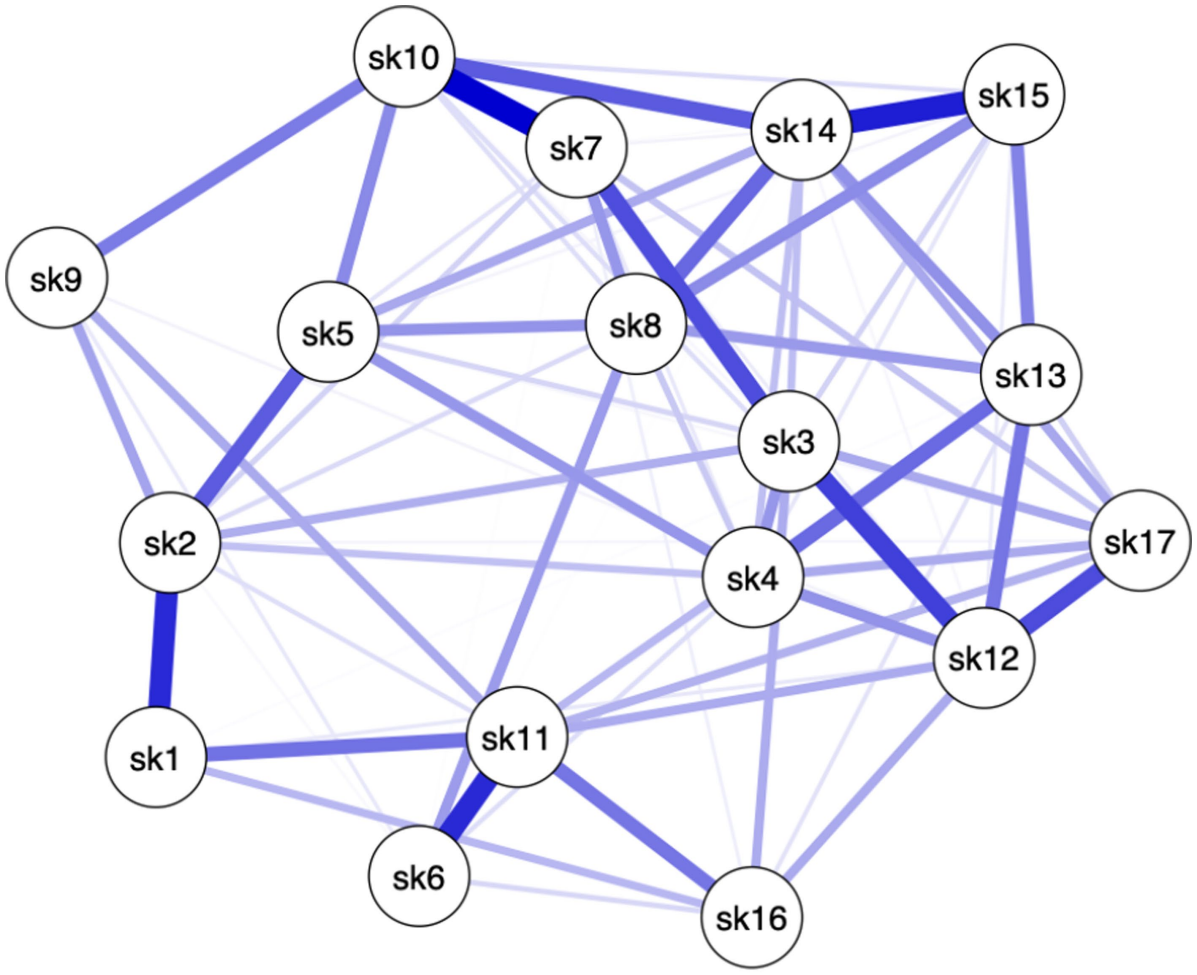
Network of Group 2: moderate HS.

**Figure 3:**
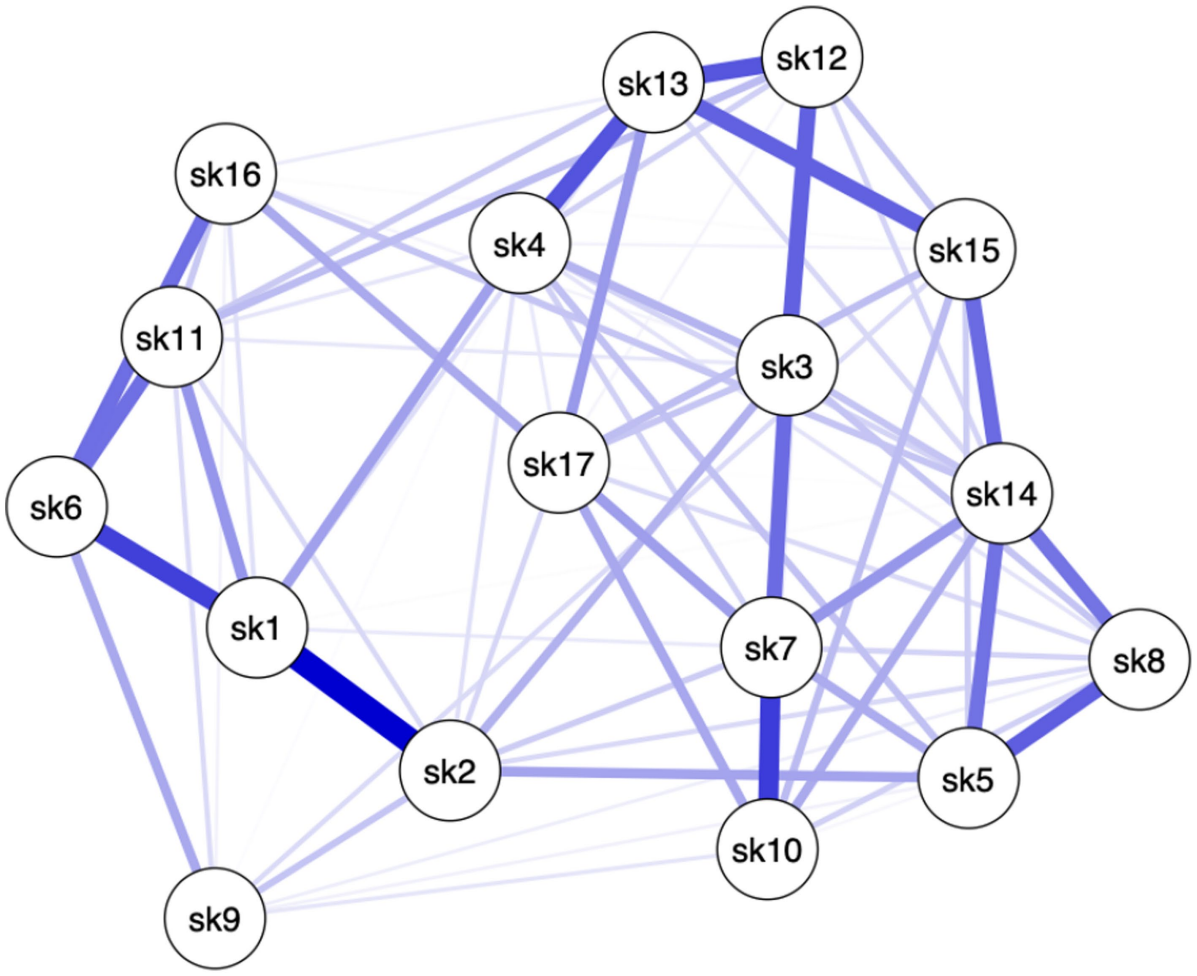
Network of Group 3: severe HS.

Different patterns among the items of the Skindex-17 emerged for the three clinical severity groups (see Figures 1–3), meaning that in each network different items are more crucial than others.

Specifically, in Group 1, paths between 11 pairs of items (8.08%, of all possible pairs) had the strongest edges (i.e., items #1–#2, items #1–#11, items #3–#7, items #3–#12, items #4–#5, items #4–#12, items #4–#13, items #6–#11, items #13–#14, items #13–#17, items #14–#15). In Group 2, paths between 9 (6.61%) pairs of items had the strongest edges (i.e., items #1–#2, items #2–#5, items #3–#7, items #3–#12, items #6–#11, items #7–#10, items #10–#14, items #12–#17, items #14–#15). In Group 3, paths between 11 (8.08%) pairs of items had the strongest edges (i.e., items #1–#2, items #1–#6, items #3–#12, items #4–#13, items #5–#8, items #6–#11, items #6–#16, items #7–#10, items #12–#13, items #13–#15, items #14–#15) (see Table 1). This means that the connection between these pairs of items, within their own network, are the strongest compared to the connection these items have with the other nodes (see Edges Weight Supplementary Table S1). In other words, these pairs of items are more connected to each other than to the rest of the items, in their respective networks. Moreover, the scores on one item can affect (or can be affected by) the score on the other item of the pair. Moreover, it is important to note that 4 pairs of items’ edges were found to be significantly the strongest in all 3 networks (i.e., items #1–#2, items #3–#12, items #6–#11, items #14–#15). This indicates that among all nodes, 4 pairs of items are associated to all levels of HS severity. In other words, these specific 4 pairs of items do not seem to be affected by changes in HS severity.

**Figure 4:**
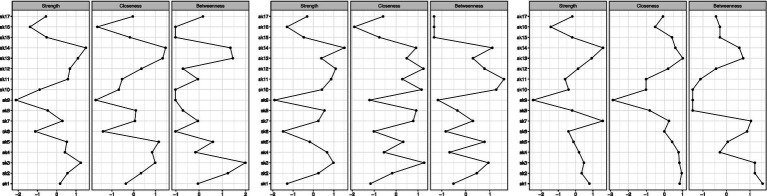
Centrality tables of Group 1, Group 2, and Group 3, respectively.

In the 3 networks, items of the Skindex-17 reported different centrality indices (see Figure 4). Specifically, in Group 1, the nodes that had the highest ***betweenness*** were items #2, #3, #13 and #14 (respectively, 1.23, 1.99, 1.34), while in Group 2 such nodes were items #10, #11 and #14 (respectively,1.26, 1.59, 1.10), and in Group 3 items #1, #2, #3 and #7 (respectively, 1.53, 1.20, 1.20, 1.04), meaning that these items have highest number of shortest paths connecting them to the other items within their networks. In other words, these items can be considered as the most important mediators to all the other items. The nodes that had the highest ***closeness*** in Group 1 were items #5, #13 and #14 (respectively, 1.15, 1.33, 1.46), while in Group 2 they were items #3, #10 and #12 (1.28, 1.16, 1.24), and in Group 3 item #13 (1.01), meaning that these items share the highest number of direct paths connecting them to the other items within their network. In other words, these items are the most associated to the majority of the other items. Finally, the nodes that had the highest ***strength*** in Group 1 were items #3, #13 and #14 (1.24, 1.09, 1.50), while in Group 2 they were items #12 and #14 (1.09, 1.52), and in Group 3 items #7 and #14 (1.57, 1.59), meaning that these items have the strongest connection to all the other items within their network. In other words, these items share the highest correlation to the items to which they are connected to.

## 4. Discussion

In our study we have depicted the specific contribution of each item of the Skindex-17, separately for mild, moderate, and severe HS. This network analysis is, to the best of our knowledge, the first one applied in HS research.

Our results highlight the connection between the items of the Skindex-17 and provide new insights on how they interact within different levels of HS severity. In other words, according different levels of HS severity, some items are more interconnected than others, more central, and have more strong interactions among all items.

Even though in all 3 groups all items have positive connections throughout the networks, several items share weak connections with others (edges strength), and only less than 10% of items connections in each network had the strongest edges. These results mean that some nodes are conditionally independent from all the other nodes (see Supplementary material for edges weights). Specifically, different sets of items showed the highest centrality values for each network (i.e., Group 1: #2, #3, #5, #13 and #14; Group 2: #3, #10, #11, #12 and #14; Group 3: #1, #2, #3, #7, #13 and #14). To interpret these findings both the statistical meaning of centrality values and nodes connections (edges) must be taken into account. For example, within Group 1, item #14 reported the highest centrality indices and one of the strongest edge strengths. This means that not only item #14 is more central than other items in the network, but also that a high score on item #14 will be associated to a high score to all the items that item #14 is strongly connected to. To obtain a more precise understanding of the association between Skindex-17 and different levels of HS severity, one must consider to which Sindex-17 subscales belong the most central items of each network. The psychosocial subscale is composed of items #2 (i.e., “*work/hobbies*”), #3 (i.e., “*social life*”), #4 (i.e., “*depressed*”), #5 (i.e., “*stay home*”), #7 (i.e., “*closeness*”), #8 (i.e., “*do things alone*”), #10 (i.e., “*show affection*”), #12 (i.e., “*embarrassed*”), #13 (i.e., “*frustrated*”), #14 (i.e., “*be with people*”), #15 (i.e., “*humiliated*”) and #17 (i.e., “*sex life*”), and the symptoms subscale is composed of items #1 (i.e., “*hurts*”), #6 (i.e., “*itches*”), #9 (i.e., “*water bothers*”), #11 (i.e., “*irritated*”) and #16 (i.e., “*bleeds*”). Considering both the content of each item and the most central items within each network, we can state that: individuals from all three groups of HS, predominantly place more importance on the items of the psychosocial subscale, than on those of the symptoms subscale. Indeed, the items that share the highest centrality indices in all three groups are included in the Psychosocial subscale of the Skindex-17. Since the centrality indices should be interpreted together, our findings seem to suggest that impairments in psychosocial aspects are more central in understanding the QoL of patients with HS, than physical symptoms. However, only one item of the symptoms subscale was found to be the central for individuals with moderate and severe HS, and that item was different for the two groups. Individuals with moderate HS only prioritized item #11 (i.e., “*irritated*”) of the symptoms subscale, while individuals with severe HS only prioritized item #1 (i.e., “*hurts*”) of the symptoms subscale.

These results indicate that, overall, the psychosocial subscale of the Skindex-17 is the most relevant when assessing the QoL of individuals with different levels of HS severity, however, with the progression of the disease, individuals place attention also on two different aspects of the symptoms subscale of the Skindex-17, irritation and pain, which in turn perfectly reflect the severity of HS, from a clinical perspective.

Furthermore, these results could explain why Sampogna et al. found that there were no significant associations between Skindex-17 and clinical severity of HS, measured with IHS4, and provide indication to how properly assess QoL of individuals with different levels of HS, with the Skindex-17 (15). Lastly, knowing which items (i.e., psychosocial and physical symptoms) are more central to each level of HS severity, might help providing more efficient interventions, from both the physical and psychological perspective.

Our results could indicate that a stronger focus on the psychosocial symptoms measured by the items that reported the highest centrality indices could be helpful for clinical intervention, since those symptoms are the most connected to the others, and if tackled could lead to quicker improvement in patients’ QoL.

Although interesting, our results are not immune to limitations. Firstly, since we used cross-sectional data to conduct the analyses, we cannot determine the direction of edges nor the presence of self-reinforcing edges. This means that we do not know whether the most important items are activated by other items, or if they activate other items. Secondly, self-report measures, as the Skindex-17, can be affected by multiple response biases, such as social desirability (31), which in turn could have affected the network stability. Thirdly, the Skindex-17 uses single-items to measure each symptom. Taken together, these limitations might impact the generalizability and robustness of our findings, in terms of adequacy of the model and stability of the parameters of the Networks.

Finally, although it can be argued that the removal of individuals with missing data for either the IHS4 and the Skindex-17 led to a reduction of sample size, which in turn could affect generalizability of the results, the inclusion of only patients with full data actually strengthens the stability of the results and reduces error estimates and biases that can arise when replacing missing data (32). Moreover, the sample size still remained high enough to perform the required analyses.

Our study, aimed at assessing the specific relationship between items of the Skindex-17 and levels of HS severity through a Network Analysis, indicates that persons with HS place more importance on the items of the psychosocial subscale of the Skindex-17, and only individuals with moderate and severe HS place importance on two different items of the symptoms subscale. These results provide new insights on the association between levels of HS severity and related QoL, measured with the Skindex-17. The findings provided in this study added new important insight into understanding how psychosocial and physical issues are experienced by patients with different levels of disease. Longitudinal studies should further the results found by the current research by analyzing whether results of the network analysis change during the course of time and by assessing the direction of the observed edges. For example, within each HS severity group, knowing which items affects the other could help clinicians to provide more *ad-hoc* interventions, since each item of the Skindex-17 measure a specific symptom (either psychosocial or physical). Therefore, this could lead to the achievement of more and quickly improvement in the Quality of Life of the patients, since the focus of psychological and physical treatment would be directed to the symptoms that “activate” the others. Finally, the identification of self-reinforcing edges could help better understand the interplay of psychosocial and physical symptoms, which in turn could lead to an improvement in the psychological treatment of these patients.

## Data availability statement

The data that support the findings of this study are available from the corresponding author, [GR], upon reasonable request.

## Ethics statement

The studies involving humans were approved by Institutional Ethical Committee of IDIIRCCS (459/1, 9 November 2015). The studies were conducted in accordance with the local legislation and institutional requirements. The participants provided their written informed consent to participate in this study.

## Author contributions

GR: Conceptualization, Data curation, Formal analysis, Methodology, Writing – original draft. TS: Methodology, Writing – original draft, Writing – review & editing. LF: Methodology, Writing – review & editing. DA: Funding acquisition, Methodology, Supervision, Writing – review & editing.
