# Supplementary material for: Item-specific patterns of the Skindex-17 in individuals with different levels of Hidradenitis Suppurativa severity: a network analysis study
Source: Front Public Health. 2023 Oct 27;11:1284365. doi: 10.3389/fpubh.2023.1284365 (PMC10646184; doi:10.3389/fpubh.2023.1284365)
Supplement: Supplementary file 1 [file Table_1.DOCX]

**Table S1**. Edges weight for the three Networks.

| **Node 1** | **Node 2** | **Edges Weights** | **Lower 95% CI** | **Upper 95% CI** |
| --- | --- | --- | --- | --- |
| *Network 1 – Mild HS* | | | | |
| sk1 | sk10 | 0.00 | -0.03 | 0.03 |
| sk1 | sk11 | 0.22 | 0.06 | 0.38 |
| sk1 | sk12 | 0.02 | -0.07 | 0.08 |
| sk1 | sk13 | 0.01 | -0.07 | 0.07 |
| sk1 | sk14 | 0.00 | -0.01 | 0.01 |
| sk1 | sk15 | -0.01 | -0.06 | 0.06 |
| sk1 | sk16 | 0.08 | -0.02 | 0.23 |
| sk1 | sk17 | 0.00 | -0.02 | 0.03 |
| sk1 | sk2 | 0.27 | 0.12 | 0.48 |
| sk1 | sk3 | 0.01 | -0.06 | 0.06 |
| sk1 | sk4 | 0.02 | -0.06 | 0.11 |
| sk1 | sk5 | 0.01 | -0.06 | 0.06 |
| sk1 | sk6 | 0.16 | 0.02 | 0.35 |
| sk1 | sk7 | -0.04 | -0.12 | 0.12 |
| sk1 | sk8 | 0.00 | -0.03 | 0.03 |
| sk1 | sk9 | 0.03 | -0.05 | 0.13 |
| sk10 | sk11 | -0.00 | -0.02 | 0.03 |
| sk10 | sk12 | 0.03 | -0.06 | 0.14 |
| sk10 | sk13 | 0.00 | -0.05 | 0.05 |
| sk10 | sk14 | 0.15 | 0.020 | 0.34 |
| sk10 | sk15 | 0.00 | -0.03 | 0.03 |
| sk10 | sk16 | -0.01 | -0.08 | 0.08 |
| sk10 | sk17 | -0.00 | -0.02 | 0.02 |
| sk11 | sk12 | 0.06 | -0.04 | 0.19 |
| sk11 | sk13 | 0.08 | -0.02 | 0.23 |
| sk11 | sk14 | -0.00 | -0.02 | 0.03 |
| sk11 | sk15 | -0.00 | -0.01 | 0.02 |
| sk11 | sk16 | 0.08 | -0.05 | 0.24 |
| sk11 | sk17 | 0.02 | -0.07 | 0.08 |
| sk12 | sk13 | 0.11 | -0.02 | 0.27 |
| sk12 | sk14 | 0.02 | -0.07 | 0.07 |
| sk12 | sk15 | 0.11 | -0.02 | 0.26 |
| sk12 | sk16 | 0.00 | -0.02 | 0.03 |
| sk12 | sk17 | 0.07 | -0.05 | 0.23 |
| sk13 | sk14 | 0.22 | 0.12 | 0.38 |
| sk13 | sk15 | 0.05 | -0.04 | 0.15 |
| sk13 | sk16 | 0.05 | -0.04 | 0.16 |
| sk13 | sk17 | 0.23 | 0.147 | 0.37 |
| sk14 | sk15 | 0.21 | 0.09 | 0.38 |
| sk14 | sk16 | 0.03 | -0.06 | 0.10 |
| sk14 | sk17 | 0.01 | -0.05 | 0.05 |
| sk15 | sk16 | 0.01 | -0.05 | 0.06 |
| sk15 | sk17 | 0.02 | -0.07 | 0.09 |
| sk16 | sk17 | 0.09 | -0.05 | 0.27 |
| sk2 | sk10 | 0.00 | -0.03 | 0.03 |
| sk2 | sk11 | 0.13 | -0.00 | 0.30 |
| sk2 | sk12 | 0.03 | -0.07 | 0.10 |
| sk2 | sk13 | 0.04 | -0.05 | 0.15 |
| sk2 | sk14 | 0.04 | -0.05 | 0.14 |
| sk2 | sk15 | -0.00 | -0.02 | 0.03 |
| sk2 | sk16 | 0.00 | -0.03 | 0.04 |
| sk2 | sk17 | 0.03 | -0.07 | 0.14 |
| sk2 | sk3 | 0.09 | -0.00 | 0.23 |
| sk2 | sk4 | 0.02 | -0.07 | 0.08 |
| sk2 | sk5 | 0.16 | 0.06 | 0.33 |
| sk2 | sk6 | 0.03 | -0.08 | 0.13 |
| sk2 | sk7 | 0.07 | -0.07 | 0.20 |
| sk2 | sk8 | 0.00 | -0.04 | 0.05 |
| sk2 | sk9 | 0.02 | -0.05 | 0.08 |
| sk3 | sk10 | 0.04 | -0.06 | 0.14 |
| sk3 | sk11 | 0.02 | -0.06 | 0.10 |
| sk3 | sk12 | 0.23 | 0.11 | 0.42 |
| sk3 | sk13 | 0.02 | -0.08 | 0.09 |
| sk3 | sk14 | 0.17 | 0.05 | 0.32 |
| sk3 | sk15 | 0.05 | -0.05 | 0.17 |
| sk3 | sk16 | -0.01 | -0.06 | 0.06 |
| sk3 | sk17 | 0.03 | -0.06 | 0.12 |
| sk3 | sk4 | 0.02 | -0.06 | 0.09 |
| sk3 | sk5 | 0.10 | -0.03 | 0.25 |
| sk3 | sk6 | 0.00 | -0.04 | 0.04 |
| sk3 | sk7 | 0.19 | 0.03 | 0.38 |
| sk3 | sk8 | 0.04 | -0.07 | 0.14 |
| sk3 | sk9 | 0.09 | 0.01 | 0.24 |
| sk4 | sk10 | 0.01 | -0.05 | 0.05 |
| sk4 | sk11 | 0.05 | -0.04 | 0.17 |
| sk4 | sk12 | 0.18 | 0.06 | 0.36 |
| sk4 | sk13 | 0.20 | 0.07 | 0.38 |
| sk4 | sk14 | 0.02 | -0.06 | 0.09 |
| sk4 | sk15 | 0.16 | 0.06 | 0.31 |
| sk4 | sk16 | 0.00 | -0.02 | 0.03 |
| sk4 | sk17 | 0.00 | -0.04 | 0.04 |
| sk4 | sk5 | 0.17 | 0.08 | 0.35 |
| sk4 | sk6 | -0.00 | -0.03 | 0.03 |
| sk4 | sk7 | 0.01 | -0.06 | 0.06 |
| sk4 | sk8 | 0.02 | -0.06 | 0.08 |
| sk4 | sk9 | 0.00 | -0.02 | 0.02 |
| sk5 | sk10 | 0.01 | -0.07 | 0.07 |
| sk5 | sk11 | 0.00 | -0.03 | 0.04 |
| sk5 | sk12 | 0.00 | -0.03 | 0.04 |
| sk5 | sk13 | 0.03 | -0.06 | 0.11 |
| sk5 | sk14 | 0.13 | -0.00 | 0.29 |
| sk5 | sk15 | 0.02 | -0.08 | 0.08 |
| sk5 | sk16 | 0.01 | -0.06 | 0.07 |
| sk5 | sk17 | 0.00 | -0.03 | 0.03 |
| sk5 | sk6 | -0.00 | -0.03 | 0.03 |
| sk5 | sk7 | 0.10 | -0.05 | 0.27 |
| sk5 | sk8 | 0.17 | 0.041 | 0.35 |
| sk5 | sk9 | 0.01 | -0.07 | 0.07 |
| sk6 | sk10 | 0.00 | -0.05 | 0.06 |
| sk6 | sk11 | 0.22 | 0.09 | 0.39 |
| sk6 | sk12 | 0.00 | -0.03 | 0.03 |
| sk6 | sk13 | -0.00 | -0.02 | 0.02 |
| sk6 | sk14 | 0.00 | -0.02 | 0.03 |
| sk6 | sk15 | 0.01 | -0.05 | 0.06 |
| sk6 | sk16 | 0.06 | -0.09 | 0.19 |
| sk6 | sk17 | 0.00 | -0.05 | 0.05 |
| sk6 | sk7 | 0.00 | -0.03 | 0.04 |
| sk6 | sk8 | -0.00 | -0.04 | 0.04 |
| sk6 | sk9 | -0.00 | -0.05 | 0.06 |
| sk7 | sk10 | 0.15 | -0.00 | 0.34 |
| sk7 | sk11 | 0.00 | -0.01 | 0.01 |
| sk7 | sk12 | 0.09 | -0.03 | 0.23 |
| sk7 | sk13 | -0.00 | -0.03 | 0.04 |
| sk7 | sk14 | 0.02 | -0.08 | 0.08 |
| sk7 | sk15 | 0.01 | -0.05 | 0.06 |
| sk7 | sk16 | -0.00 | -0.03 | 0.03 |
| sk7 | sk17 | 0.12 | -0.00 | 0.28 |
| sk7 | sk8 | 0.11 | -0.02 | 0.28 |
| sk7 | sk9 | -0.00 | -0.03 | 0.03 |
| sk8 | sk10 | 0.13 | -0.04 | 0.33 |
| sk8 | sk11 | 0.01 | -0.06 | 0.07 |
| sk8 | sk12 | 0.00 | -0.03 | 0.03 |
| sk8 | sk13 | 0.01 | -0.06 | 0.06 |
| sk8 | sk14 | 0.16 | 0.03 | 0.34 |
| sk8 | sk15 | 0.01 | -0.07 | 0.07 |
| sk8 | sk16 | -0.00 | -0.03 | 0.04 |
| sk8 | sk17 | 0.00 | -0.03 | 0.04 |
| sk8 | sk9 | 0.01 | -0.08 | 0.08 |
| sk9 | sk10 | 0.03 | -0.11 | 0.12 |
| sk9 | sk11 | 0.03 | -0.06 | 0.12 |
| sk9 | sk12 | -0.00 | -0.02 | 0.02 |
| sk9 | sk13 | 0.00 | -0.04 | 0.04 |
| sk9 | sk14 | -0.00 | -0.04 | 0.04 |
| sk9 | sk15 | -0.00 | -0.04 | 0.05 |
| sk9 | sk16 | 0.02 | -0.09 | 0.09 |
| sk9 | sk17 | -0.0 | -0.05 | 0.05 |
| *Network 2 – Moderate HS* | | | | |
| sk1 | sk10 | -0.04 | -0.04 | 0.05 |
| sk1 | sk11 | 0.16 | 0.04 | 0.31 |
| sk1 | sk12 | 0.03 | -0.05 | 0.10 |
| sk1 | sk13 | 0.02 | -0.06 | 0.07 |
| sk1 | sk14 | -0.01 | -0.01 | 0.01 |
| sk1 | sk15 | -0.07 | -0.07 | 0.08 |
| sk1 | sk16 | 0.08 | -0.03 | 0.21 |
| sk1 | sk17 | 0.00 | -0.04 | 0.04 |
| sk1 | sk2 | 0.25 | 0.13 | 0.41 |
| sk1 | sk3 | 0.00 | -0.04 | 0.04 |
| sk1 | sk4 | 0.00 | -0.02 | 0.03 |
| sk1 | sk5 | 0.00 | -0.02 | 0.03 |
| sk1 | sk6 | 0.01 | -0.06 | 0.06 |
| sk1 | sk7 | -0.02 | -0.02 | 0.03 |
| sk1 | sk8 | -0.02 | -0.02 | 0.02 |
| sk1 | sk9 | 0.02 | -0.08 | 0.08 |
| sk10 | sk11 | 0.00 | -0.01 | 0.02 |
| sk10 | sk12 | 0.03 | -0.03 | 0.11 |
| sk10 | sk13 | 0.00 | -0.02 | 0.02 |
| sk10 | sk14 | 0.19 | 0.08 | 0.32 |
| sk10 | sk15 | 0.04 | -0.05 | 0.13 |
| sk10 | sk16 | -0.02 | -0.02 | 0.03 |
| sk10 | sk17 | 0.00 | -0.03 | 0.03 |
| sk11 | sk12 | 0.10 | -0.01 | 0.22 |
| sk11 | sk13 | 0.07 | -0.01 | 0.19 |
| sk11 | sk14 | 0.00 | -0.02 | 0.03 |
| sk11 | sk15 | -0.03 | -0.03 | 0.03 |
| sk11 | sk16 | 0.16 | 0.04 | 0.29 |
| sk11 | sk17 | 0.07 | -0.01 | 0.20 |
| sk12 | sk13 | 0.15 | 0.04 | 0.30 |
| sk12 | sk14 | 0.02 | -0.05 | 0.08 |
| sk12 | sk15 | 0.02 | -0.04 | 0.09 |
| sk12 | sk16 | 0.08 | -0.01 | 0.22 |
| sk12 | sk17 | 0.21 | 0.09 | 0.36 |
| sk13 | sk14 | 0.12 | 0.02 | 0.25 |
| sk13 | sk15 | 0.14 | 0.04 | 0.26 |
| sk13 | sk16 | 0.03 | -0.05 | 0.12 |
| sk13 | sk17 | 0.05 | -0.04 | 0.16 |
| sk14 | sk15 | 0.27 | 0.16 | 0.41 |
| sk14 | sk16 | 0.00 | -0.03 | 0.03 |
| sk14 | sk17 | 0.09 | 0.00 | 0.21 |
| sk15 | sk16 | 0.00 | -0.02 | 0.03 |
| sk15 | sk17 | 0.00 | -0.02 | 0.02 |
| sk16 | sk17 | 0.01 | -0.06 | 0.06 |
| sk2 | sk10 | 0.00 | -0.03 | 0.03 |
| sk2 | sk11 | 0.04 | -0.05 | 0.13 |
| sk2 | sk12 | 0.00 | -0.03 | 0.03 |
| sk2 | sk13 | 0.00 | -0.02 | 0.03 |
| sk2 | sk14 | 0.00 | -0.02 | 0.02 |
| sk2 | sk15 | -0.02 | -0.02 | 0.03 |
| sk2 | sk16 | -0.02 | -0.02 | 0.03 |
| sk2 | sk17 | 0.02 | -0.05 | 0.08 |
| sk2 | sk3 | 0.08 | -0.01 | 0.20 |
| sk2 | sk4 | 0.07 | -0.02 | 0.18 |
| sk2 | sk5 | 0.19 | 0.07 | 0.33 |
| sk2 | sk6 | 0.02 | -0.06 | 0.08 |
| sk2 | sk7 | 0.05 | -0.04 | 0.15 |
| sk2 | sk8 | 0.04 | -0.04 | 0.13 |
| sk2 | sk9 | 0.09 | -0.01 | 0.24 |
| sk3 | sk10 | 0.03 | -0.06 | 0.09 |
| sk3 | sk11 | 0.00 | -0.02 | 0.03 |
| sk3 | sk12 | 0.23 | 0.11 | 0.37 |
| sk3 | sk13 | 0.00 | -0.03 | 0.04 |
| sk3 | sk14 | 0.06 | -0.01 | 0.17 |
| sk3 | sk15 | 0.04 | -0.03 | 0.14 |
| sk3 | sk16 | 0.08 | -0.00 | 0.20 |
| sk3 | sk17 | 0.10 | -0.03 | 0.24 |
| sk3 | sk4 | 0.12 | 0.015 | 0.24 |
| sk3 | sk5 | 0.04 | -0.04 | 0.15 |
| sk3 | sk6 | -0.06 | -0.06 | 0.06 |
| sk3 | sk7 | 0.20 | 0.10 | 0.34 |
| sk3 | sk8 | 0.02 | -0.06 | 0.07 |
| sk3 | sk9 | 0.00 | -0.02 | 0.03 |
| sk4 | sk10 | 0.00 | -0.01 | 0.01 |
| sk4 | sk11 | 0.03 | -0.05 | 0.10 |
| sk4 | sk12 | 0.12 | 0.09 | 0.26 |
| sk4 | sk13 | 0.17 | 0.06 | 0.31 |
| sk4 | sk14 | 0.07 | -0.02 | 0.18 |
| sk4 | sk15 | 0.03 | -0.04 | 0.11 |
| sk4 | sk16 | 0.01 | -0.04 | 0.04 |
| sk4 | sk17 | 0.09 | -0.01 | 0.22 |
| sk4 | sk5 | 0.11 | 0.00 | 0.25 |
| sk4 | sk6 | 0.03 | -0.04 | 0.12 |
| sk4 | sk7 | 0.03 | -0.04 | 0.10 |
| sk4 | sk8 | 0.06 | -0.04 | 0.18 |
| sk4 | sk9 | 0.00 | -0.01 | 0.02 |
| sk5 | sk10 | 0.13 | 0.01 | 0.27 |
| sk5 | sk11 | -0.02 | -0.02 | 0.03 |
| sk5 | sk12 | -113 | -0.01 | 0.01 |
| sk5 | sk13 | 0.01 | -0.05 | 0.05 |
| sk5 | sk14 | 0.09 | -0.02 | 0.23 |
| sk5 | sk15 | 0.03 | -0.06 | 0.09 |
| sk5 | sk16 | 0.00 | -0.02 | 0.03 |
| sk5 | sk17 | 0.02 | -0.06 | 0.09 |
| sk5 | sk6 | 0.00 | -0.04 | 0.05 |
| sk5 | sk7 | 0.03 | -0.05 | 0.12 |
| sk5 | sk8 | 0.12 | 0.01 | 0.25 |
| sk5 | sk9 | -0.03 | -0.03 | 0.04 |
| sk6 | sk10 | -0.01 | -0.01 | 0.02 |
| sk6 | sk11 | 0.25 | 0.13 | 0.41 |
| sk6 | sk12 | -0.02 | -0.02 | 0.03 |
| sk6 | sk13 | -0.03 | -0.03 | 0.03 |
| sk6 | sk14 | 0.01 | -0.04 | 0.05 |
| sk6 | sk15 | 0.00 | -0.03 | 0.04 |
| sk6 | sk16 | 0.05 | -0.05 | 0.15 |
| sk6 | sk17 | 0.00 | -0.02 | 0.03 |
| sk6 | sk7 | 0.01 | -0.05 | 0.06 |
| sk6 | sk8 | 0.09 | 0.02 | 0.22 |
| sk6 | sk9 | 0.03 | -0.06 | 0.12 |
| sk7 | sk10 | 0.29 | 0.19 | 0.44 |
| sk7 | sk11 | 0.00 | -0.02 | 0.02 |
| sk7 | sk12 | 0.02 | -0.05 | 0.10 |
| sk7 | sk13 | 0.01 | -0.05 | 0.06 |
| sk7 | sk14 | 0.02 | -0.06 | 0.09 |
| sk7 | sk15 | 0.02 | -0.07 | 0.08 |
| sk7 | sk16 | 0.02 | -0.05 | 0.09 |
| sk7 | sk17 | 0.06 | -0.02 | 0.18 |
| sk7 | sk8 | 0.10 | 0.01 | 0.25 |
| sk7 | sk9 | 0.00 | -0.03 | 0.04 |
| sk8 | sk10 | 0.05 | -0.07 | 0.15 |
| sk8 | sk11 | 0.01 | -0.05 | 0.05 |
| sk8 | sk12 | -0.01 | -0.01 | 0.01 |
| sk8 | sk13 | 0.11 | -0.00 | 0.25 |
| sk8 | sk14 | 0.18 | 0.05 | 0.32 |
| sk8 | sk15 | 0.13 | 0.01 | 0.28 |
| sk8 | sk16 | 0.01 | -0.05 | 0.05 |
| sk8 | sk17 | -0.02 | -0.01 | 0.01 |
| sk8 | sk9 | 0.00 | -0.02 | 0.02 |
| sk9 | sk10 | 0.14 | 0.02 | 0.30 |
| sk9 | sk11 | 0.08 | 0.01 | 0.20 |
| sk9 | sk12 | 0.01 | -0.04 | 0.07 |
| sk9 | sk13 | 0.00 | -0.03 | 0.03 |
| sk9 | sk14 | 0.01 | -0.05 | 0.06 |
| sk9 | sk15 | -0.04 | -0.04 | 0.04 |
| sk9 | sk16 | -0.03 | -0.03 | 0.03 |
| sk9 | sk17 | 0.01 | -0.05 | 0.05 |
| *Network 3 – Severe HS* | | | | |
| sk1 | sk10 | 0.00 | -0.02 | 0.03 |
| sk1 | sk11 | 0.14 | 0.02 | 0.28 |
| sk1 | sk12 | 0.01 | -0.05 | 0.06 |
| sk1 | sk13 | 0.04 | -0.02 | 0.12 |
| sk1 | sk14 | 0.01 | -0.03 | 0.05 |
| sk1 | sk15 | -0.00 | -0.00 | 0.01 |
| sk1 | sk16 | 0.04 | -0.04 | 0.13 |
| sk1 | sk17 | 0.00 | -0.03 | 0.03 |
| sk1 | sk2 | 0.34 | 0.27 | 0.46 |
| sk1 | sk3 | 0.00 | -0.02 | 0.02 |
| sk1 | sk4 | 0.12 | 0.03 | 0.23 |
| sk1 | sk5 | 0.00 | -0.03 | 0.03 |
| sk1 | sk6 | 0.26 | 0.16 | 0.38 |
| sk1 | sk7 | 0.02 | -0.02 | 0.08 |
| sk1 | sk8 | 0.00 | -0.01 | 0.01 |
| sk1 | sk9 | 0.00 | -0.02 | 0.03 |
| sk10 | sk11 | 0.00 | -0.01 | 0.02 |
| sk10 | sk12 | 0.04 | -0.04 | 0.12 |
| sk10 | sk13 | -0.00 | -0.01 | 0.02 |
| sk10 | sk14 | 0.11 | 0.00 | 0.23 |
| sk10 | sk15 | 0.08 | -0.02 | 0.21 |
| sk10 | sk16 | 0.00 | -0.02 | 0.02 |
| sk10 | sk17 | 0.11 | 0.02 | 0.23 |
| sk11 | sk12 | 0.08 | -0.00 | 0.20 |
| sk11 | sk13 | 0.06 | -0.01 | 0.17 |
| sk11 | sk14 | -0.00 | -0.02 | 0.02 |
| sk11 | sk15 | -0.00 | -0.01 | 0.02 |
| sk11 | sk16 | 0.07 | -0.02 | 0.18 |
| sk11 | sk17 | 0.00 | -0.02 | 0.02 |
| sk12 | sk13 | 0.22 | 0.13 | 0.36 |
| sk12 | sk14 | 0.05 | -0.02 | 0.14 |
| sk12 | sk15 | 0.07 | -0.00 | 0.17 |
| sk12 | sk16 | 0.02 | -0.04 | 0.10 |
| sk12 | sk17 | 0.03 | -0.06 | 0.10 |
| sk13 | sk14 | 0.04 | -0.04 | 0.12 |
| sk13 | sk15 | 0.21 | 0.12 | 0.33 |
| sk13 | sk16 | 0.00 | -0.02 | 0.02 |
| sk13 | sk17 | 0.12 | 0.04 | 0.24 |
| sk14 | sk15 | 0.19 | 0.10 | 0.32 |
| sk14 | sk16 | 0.06 | -0.00 | 0.17 |
| sk14 | sk17 | 0.02 | -0.06 | 0.06 |
| sk15 | sk16 | 0.02 | -0.05 | 0.06 |
| sk15 | sk17 | 0.08 | -0.01 | 0.19 |
| sk16 | sk17 | 0.10 | 0.02 | 0.22 |
| sk2 | sk10 | 0.00 | -0.02 | 0.03 |
| sk2 | sk11 | 0.05 | -0.04 | 0.15 |
| sk2 | sk12 | -0.00 | -0.01 | 0.02 |
| sk2 | sk13 | 0.00 | -0.03 | 0.04 |
| sk2 | sk14 | 0.00 | -0.01 | 0.02 |
| sk2 | sk15 | 0.00 | -0.01 | 0.01 |
| sk2 | sk16 | 0.00 | -0.04 | 0.04 |
| sk2 | sk17 | 0.05 | -0.03 | 0.15 |
| sk2 | sk3 | 0.10 | -0.00 | 0.22 |
| sk2 | sk4 | 0.05 | -0.04 | 0.13 |
| sk2 | sk5 | 0.11 | 0.02 | 0.24 |
| sk2 | sk6 | 0.00 | -0.02 | 0.02 |
| sk2 | sk7 | 0.06 | -0.02 | 0.16 |
| sk2 | sk8 | 0.05 | -0.04 | 0.15 |
| sk2 | sk9 | 0.06 | -0.01 | 0.18 |
| sk3 | sk10 | 0.04 | -0.04 | 0.13 |
| sk3 | sk11 | 0.03 | -0.04 | 0.11 |
| sk3 | sk12 | 0.21 | 0.12 | 0.33 |
| sk3 | sk13 | 0.01 | -0.04 | 0.05 |
| sk3 | sk14 | 0.08 | -0.02 | 0.19 |
| sk3 | sk15 | -0.00 | -0.01 | 0.02 |
| sk3 | sk16 | 0.02 | -0.04 | 0.08 |
| sk3 | sk17 | 0.07 | -0.02 | 0.18 |
| sk3 | sk4 | 0.10 | 0.01 | 0.20 |
| sk3 | sk5 | 0.01 | -0.05 | 0.05 |
| sk3 | sk6 | -0.01 | -0.04 | 0.05 |
| sk3 | sk7 | 0.20 | 0.10 | 0.31 |
| sk3 | sk8 | 0.07 | -0.02 | 0.19 |
| sk3 | sk9 | -0.00 | -0.03 | 0.04 |
| sk4 | sk10 | 0.00 | -0.01 | 0.01 |
| sk4 | sk11 | 0.04 | -0.05 | 0.12 |
| sk4 | sk12 | 0.06 | -0.04 | 0.17 |
| sk4 | sk13 | 0.23 | 0.14 | 0.35 |
| sk4 | sk14 | 0.05 | -0.02 | 0.14 |
| sk4 | sk15 | 0.02 | -0.04 | 0.08 |
| sk4 | sk16 | 0.00 | -0.02 | 0.03 |
| sk4 | sk17 | 0.03 | -0.04 | 0.11 |
| sk4 | sk5 | 0.08 | -0.01 | 0.19 |
| sk4 | sk6 | -0.00 | -0.03 | 0.03 |
| sk4 | sk7 | 0.05 | -0.01 | 0.15 |
| sk4 | sk8 | 0.04 | -0.04 | 0.12 |
| sk4 | sk9 | 0.01 | -0.03 | 0.05 |
| sk5 | sk10 | 0.02 | -0.06 | 0.09 |
| sk5 | sk11 | -0.00 | -0.01 | 0.02 |
| sk5 | sk12 | 0.00 | -0.02 | 0.02 |
| sk5 | sk13 | -0.00 | -0.01 | 0.02 |
| sk5 | sk14 | 0.19 | 0.07 | 0.32 |
| sk5 | sk15 | 0.06 | -0.02 | 0.16 |
| sk5 | sk16 | -0.00 | -0.03 | 0.03 |
| sk5 | sk17 | 0.01 | -0.05 | 0.05 |
| sk5 | sk6 | -0.00 | -0.02 | 0.02 |
| sk5 | sk7 | 0.10 | 0.00 | 0.23 |
| sk5 | sk8 | 0.22 | 0.11 | 0.35 |
| sk5 | sk9 | 0.02 | -0.04 | 0.09 |
| sk6 | sk10 | 0.00 | -0.02 | 0.02 |
| sk6 | sk11 | 0.19 | 0.08 | 0.32 |
| sk6 | sk12 | 0.00 | -0.02 | 0.03 |
| sk6 | sk13 | 0.00 | -0.03 | 0.04 |
| sk6 | sk14 | 0.00 | -0.01 | 0.02 |
| sk6 | sk15 | 0.00 | -0.02 | 0.02 |
| sk6 | sk16 | 0.19 | 0.09 | 0.32 |
| sk6 | sk17 | -0.00 | -0.03 | 0.03 |
| sk6 | sk7 | 0.00 | -0.03 | 0.04 |
| sk6 | sk8 | 0.00 | -0.02 | 0.03 |
| sk6 | sk9 | 0.10 | 0.03 | 0.21 |
| sk7 | sk10 | 0.26 | 0.17 | 0.40 |
| sk7 | sk11 | 0.00 | -0.02 | 0.03 |
| sk7 | sk12 | 0.06 | -0.02 | 0.16 |
| sk7 | sk13 | 0.01 | -0.04 | 0.05 |
| sk7 | sk14 | 0.13 | 0.03 | 0.26 |
| sk7 | sk15 | 0.01 | -0.06 | 0.06 |
| sk7 | sk16 | 0.02 | -0.04 | 0.07 |
| sk7 | sk17 | 0.12 | 0.03 | 0.24 |
| sk7 | sk8 | 0.06 | -0.04 | 0.16 |
| sk7 | sk9 | 0.01 | -0.04 | 0.05 |
| sk8 | sk10 | 0.06 | -0.03 | 0.16 |
| sk8 | sk11 | 0.00 | -0.02 | 0.03 |
| sk8 | sk12 | 0.00 | -0.02 | 0.02 |
| sk8 | sk13 | 0.04 | -0.02 | 0.13 |
| sk8 | sk14 | 0.18 | 0.08 | 0.31 |
| sk8 | sk15 | 0.01 | -0.05 | 0.06 |
| sk8 | sk16 | -0.00 | -0.03 | 0.04 |
| sk8 | sk17 | 0.04 | -0.03 | 0.14 |
| sk8 | sk9 | 0.02 | -0.04 | 0.10 |
| sk9 | sk10 | 0.02 | -0.03 | 0.11 |
| sk9 | sk11 | 0.04 | -0.02 | 0.12 |
| sk9 | sk12 | 0.00 | -0.03 | 0.03 |
| sk9 | sk13 | 0.00 | -0.02 | 0.03 |
| sk9 | sk14 | 0.01 | -0.04 | 0.04 |
| sk9 | sk15 | 0.03 | -0.03 | 0.13 |
| sk9 | sk16 | 0.02 | -0.05 | 0.10 |
| sk9 | sk17 | 0.00 | -0.03 | 0.03 |
